# Pharmacokinetics of L-theanine and the effect on amino acid composition in mice administered with L-theanine

**DOI:** 10.1007/s00726-024-03389-3

**Published:** 2024-04-07

**Authors:** Shinnosuke Yamaura, Koki Sadamori, Reiko Konishi, Takashi Majima, Akira Mukai, Kyosuke Uno, Toshihiko Kinjo, Koji Komori, Nobuyuki Kuramoto, Kou Kawada

**Affiliations:** 1 Laboratory of Clinical Pharmacology and Therapeutics, Faculty of Pharmaceutical Sciences, Setsunan University, Hirakata, Osaka Japan; 2 Department of Bioactive Molecules, Pharmacology, Gifu Pharmaceutical University, Gifu, Japan; 3 Department of Nursing, Faculty of Allied Health Sciences, Yamato University, Suita, Osaka Japan; 4 Laboratory of Molecular Pharmacology, Faculty of Pharmaceutical Sciences, Setsunan University, Hirakata, Osaka Japan

**Keywords:** L-theanine, Supplement, Phenylisothiocyanate, Amino acid composition, Glycine

## Abstract

L-theanine, an amino acid component of the tea leaves of *Camellia sinensis*, is sold in Japan as a supplement for good sleep. Although several studies in humans and mice have reported the effects of L-theanine on brain function, only a few reports have comprehensively clarified the disposition of theanine administered to mice and its effects on concentrations of other blood amino acids. In this study, we aimed to determine the changes in the blood levels of L-theanine administered to mice and amino acid composition of the serum. L-theanine were administered to four-week-old Std-ddY male mice orally or via tail vein injection. L-theanine and other amino acids in serum prepared from blood collected at different time points post-dose were labeled with phenylisothiocyanate and quantified. The serum concentration of orally administered L-theanine peaked 15 min after administration. The area under the curve for tail vein injection revealed the bioavailability of L- theanine to be approximately 70%. L-theanine administration did not affect any amino acid levels in the serum, but a significant increase in the peak area overlapping the Glycine (Gly) peak was observed 30 min after administration. L-theanine administered to mice was rapidly absorbed and eliminated, suggesting that taking L-theanine as a supplement is safe without affecting its own levels or serum levels of other amino acids. However, considering that Gly, similar to L-theanine, is used as a dietary supplement for its anxiolytic effects and to improve sleep, determining the effects of L-theanine administration on Gly is important and needs further research.

## Introduction

Green tea made from the leaves of the tea plant *Camellia sinensis* is consumed on a daily basis worldwide. Research on the health promoting components of green tea has been actively conducted for several years. L-theanine is a non-protein amino acid and the umami component of green tea. It is synthesized from glutamic acid (Glu) and ethylamine in the roots of tea plants, moves to the leaves, and is converted to tannin by the ultraviolet rays of sunlight. In vivo, L-theanine, when ingested as a beverage, exists mainly in its free form and is metabolized to Glu and ethylamine by glutaminases (Tsuge et al. 2003). L-theanine has many beneficial effects, such as antioxidant, anticancer, and anti-inflammatory effects, as well as protection of the cranial nerves (Li et al. 2022; Deng et al. 2016; Zhao et al. 2020). Additionally, L-theanine exhibits anti-anxiety effects and improves sleep quality (Jang et al. 2012). In Japan, isolated L-theanine is sold as a supplement, claiming its stress-relieving and relaxing effects.

Many studies have tested the effects of L-theanine on brain function in mice to elucidate its pharmacological effects. For example, a study in mice reported that L-theanine inhibits stress-induced brain atrophy (Unno et al. 2020), increases the levels of acetylcholine and γ-aminobutyric acid (GABA) in the brain, and decreases serotonin (5-HT) levels (Zhang et al. 2021). However, no studies have clarified the kinetic parameters of L-theanine in mice, such as systemic clearance, total body clearance (CLtot), volume of distribution (Vd), area under the blood concentration–time curve (AUC), and bioavailability. One reason for this may be that because L-theanine is an amino acid, the separation and quantification of L-theanine and other amino acids from biological samples have not been sufficiently studied. Therefore, in this study, we measured the concentration of L-theanine administered orally or via tail vein injection to mice using an amino acid analytical method, the phenylisothiocyanate (PITC) method, and analyzed its pharmacokinetics and effects on the composition of other serum amino acids such as Glu.

## Materials and methods

### Experimental reagents

L-theanine (Taiyo Kagaku Co., Ltd., Yokkaichi, Mie, Japan) was purchased. Amino acid standard solution H-type mixture, ornithine (Orn), citrulline (Cit), GABA, taurine (Tau), β-hydroxyproline (β-Hyp), and tryptophan (Trp) were used as standards for identifying amino acids in serum,; PITC was used as a labeling reagent, and mobile phases known as Phenylthiocarbamide (PTC) Amino Acid Mobile Phase A (water/acetonitrile = 94/6) and B (water/acetonitrile = 40/60) were purchased from FUJIFILM Wako Pure Chemical Corporation (Osaka, Japan). All the other reagents were of standard grade or higher.

### Animals

Forty-two 4-week-old mice weighing 20–30 g (SHIMIZU Laboratory Supplies Co., Ltd., Kyoto, Japan) were purchased. The mice were maintained under the following conditions: temperature, 22 ± 2 °C; humidity, 55 ± 5%; light–dark cycle, 12-h, and had free access to food and water. This study was approved by the Institutional Animal Care Committee of the Setsunan University (K21-28 and K22-27).

### Collection and preparation of serum as a sample

Mice were divided into a control group of 6 mice and treatment group of 36 mice and fasted for 12 h before starting the experiments. The control group was administered saline, and the treatment group was administered L-theanine at doses of 100, 400, and 1000 mg/kg bw orally or via tail vein injection. Blood samples (50 µL) were collected from the tail vein at 0, 15, 30, 60, and 120 min after administration. The collected blood was immediately transferred to ice for 45–60 min. The samples were then centrifuged (1000 × *g*) at 4 °C for 10 min, and 10 µL of the supernatant as the serum sample was used for analysis.

### Analytical conditions for amino acids

The HPLC system consisted of a JASCO PU-2080 Plus HPLC Pump (JASCO Corporation, Hachioji, Tokyo, Japan), SPD-10A UV–Vis detector (SHIMADZU Corporation, Kyoto, Japan), and CTO-10A column oven (SHIMADZU Corporation, Kyoto, Japan). A Wakosil-PTC (4.0 × 200 mm) column (FUJIFILM Wako Pure Chemical Corporation, Osaka, Japan) was used at 40 °C. PTC-amino acid mobile phases A and B (FUJIFILM Wako Pure Chemical Corporation, Osaka, Japan) were run in a linear gradient mode so that the proportion of mobile phase B was 70% in 15 min, and the flow rate was 1 mL/min. The detection wavelength was set at 254 nm and the sample injection volume at 10 µL.

### Preparation of samples for analysis

Amino acid analysis was performed according to the PITC method. Briefly, 10 μL of the sample was dried under reduced pressure, dissolved in 20 μL of a mixture of ethanol/water/triethylamine (TEA) (2/2/1), and dried again under reduced pressure. Further, 20 μL of a mixed solution of ethanol/water/TEA/PITC (7/1/1/1) was added, allowed to react at room temperature (20–25 °C) for 20 min, and dried under reduced pressure. Thereafter, the dry matter was dissolved in 1 mL mobile phase A and used for analysis.

### Amino acid analysis and quantification

For all the amino acids, including L-Theanine, Aspartic Acid (Asp), Glu, β-Hyp, Serine (Ser), Glutamine (Gln), Glycine (Gly), β- alanine (β-Ala), GABA, Cit, Tau, Threonine (Thr), Alanine (Ala), Proline (Pro), Tyrosine (Tyr), Valine (Val), Methionine (Met), Histidine (His), Cysteine (Cys), Arginine (Arg), Isoleucine (Ile), Leucine (Leu), Phenylalanine (Phe), Orn, Trp, and Lysine (Lys), standard samples were used to evaluate the degree of separation. The separation performance of each amino acid was evaluated as “good” and “poor” when the degree of separation was ≥ 1.5 and < 1.5, respectively. The concentration of each amino acid was calculated as the ratio of the peak area to that of the standard sample. Particularly, L-theanine concentration was calculated from a calibration curve prepared from 1.7 (0.01), 8.7 (0.05), 17.4 (0.1), 87.1 (0.5), 174.2 (1.0), 871.0 (5.0), and 1742.0 (10.0) µg/mL (mM) standard samples, and daily fluctuations were evaluated. The obtained concentrations of the two groups were compared and statistically evaluated using the Wilcoxon rank sum test. p < 0.05 was considered statistically significant.

## Results

### Chromatogram and retention time of each amino acid

**Fig. 1:**
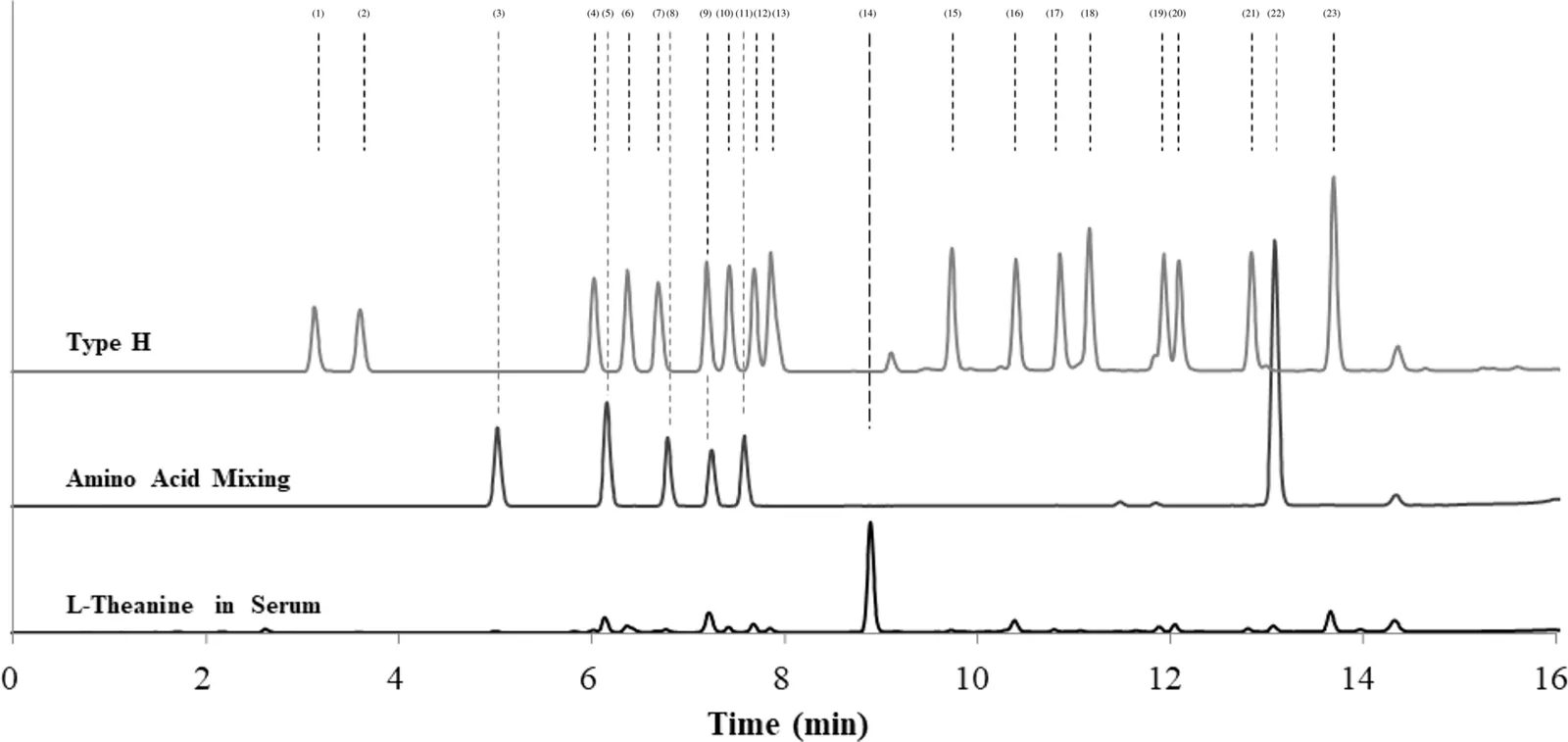
The chromatograms of H-type standards, amino acid mixtures, and serum samples processed and analyzed using PITC method. Amino acids in the order of retention time are (1) Asp, (2) Glu, (3) β-Hyp, (4) Ser, (5) Gln, (6) Gly, (7) His, (8) Cit, (9) Tau and Arg, (10) Thr, (11) GABA, (12) Ala, (13) Pro, (14) L-theanine, (15) Tyr, (16) Val, (17) Met, (18) Cys, (19) Ile, (20) Leu, (21) Phe, (22) Orn and Trp, and (23) Lys

Of the 23 amino acids, including the Type H standard, Tau and Arg and Orn and Trp could not be separated (Fig. 1). The average peak retention time (min) of each amino acid was 3.11 ± 0.01 for Asp, 3.56 ± 0.02 for Glu, 4.97 ± 0.02 for β-Hyp, 6.00 ± 0.02 for Ser, 6.11 ± 0.02 for Gln, 6.35 ± 0.02 for Gly, 6.64 ± 0.02 for His, 6.74 ± 0.02 for Cit, 7.21 ± 0.02 for Tau and Arg, 7.41 ± 0.02 for Thr, 7.57 ± 0.01 for GABA, 7.67 ± 0.02 for Ala, 7.84 ± 0.02 for Pro, 8.87 ± 0.02 for L-Theanine, 9.72 ± 0.03 for Tyr, 10.39 ± 0.03 for Val, 10.80 ± 0.03 for Met, 11.08 ± 0.03 for Cys, 11.90 ± 0.03 for Ile, 12.06 ± 0.03 for Leu, 12.82 ± 0.03 for Phe, 13.08 ± 0.03 for Orn and Trp, and 13.68 ± 0.04 for Lys.

The separation performance of Asp, Glu, β-Hyp, Gly, Tyr, Val, Met, Cys, Phe, and Lys was “good,” whereas it was “poor” for Ser, Gln, His, Cit, Thr, GABA, Ala, Pro, Ile, and Leu. Asp, with a concentration of 1.03 ± 0.24 µg/mL, was the least abundant amino acid in the serum of mice that were not administered L-theanine. The most abundant amino acid was Gln at 74.81 ± 7.50 µg/mL, followed by Lys at 72.63 ± 5.12 µg/mL. Other relatively abundant amino acids were Gly, Thr, Ala, Tyr, Val, Ile, Leu, and Phe at approximately 10–25 µg/mL, and Glu, β-Hyp, Ser, His, Cit, Pro, Met, and Cys at approximately 4–9 µg/mL. GABA was not detected (Table 1).

**Table 1:** Mean serum amino acid concentrations from 0 to 120 min in mice treated with saline and L-Theanine, and evaluation of the isolation of each amino acid

	Amino acid		Retention time (min)				Quantitativity^a^				0–120 min n = 6 Concentration in control group (µg/mL)			
(1)	D	Asp		3.11	±	0.01	> ** > ** ** > ** ** > ** ** > ** ** > ** ** > **		Good Good Good Poor Good Good Poor		1.03	±	0.24	
(2)	E	Glu		3.56	±	0.02					5.09	±	1.68	
(3)	β-H	β-Hyp		4.97	±	0.02					6.48	±	0.26	
(4)	S	Ser		6.00	±	0.02					7.16	±	0.76	
(5)	Q	Gln		6.11	±	0.02					74.81	±	7.50	
(6)	G	Gly		6.35	±	0.02					16.59	±	1.68	
(7)	H	His		6.64	±	0.02					9.33	±	0.68	
(8)	Ci	Cit		6.74	±	0.02					7.65	±	0.32	
(9)	Ta R	Tau Arg		7.21	±	0.02		Not separated			—			
(10)	T	Thr		7.41	±	0.02	** > ** ** > ** ** > ** ** > ** ** > ** ** > ** ** > ** ** > ** ** > ** ** > ** ** > **		Poor Poor Poor **Good** **Good** Good Good Good Good Poor Good		15.66	±	1.14	
(11)	G-A	GAB		7.57	±	0.01					not detected			
(12)	A	Ala		7.67	±	0.02					19.02	±	1.35	
(13)	P	Pro		7.84	±	0.02					9.20	±	0.51	
(14)	**L-Theanine**			**8.87**	** ± **	**0.02**					not detected			
(15)	Y	Tyr		9.72	±	0.03					11.02	±	1.14	
(16)	V	Val		10.39	±	0.03					24.05	±	2.50	
(17)	M	Met		10.80	±	0.03					6.38	±	0.46	
(18)	C	Cys		11.08	±	0.03					3.80	±	0.43	
(19)	I	Ile		11.90	±	0.03					11.67	±	1.52	
(20)	L	Leu		12.06	±	0.03					20.70	±	2.25	
(21)	F	Phe		12.82	±	0.03					14.24	±	1.21	
(22)	Or W	Orn Trp		13.08	±	0.03		Not separated			—			
(23)	K	Lys		13.68	±	0.04					72.63	±	5.12	

^a^ Separability was rated as “good” when the degree of separation was ≥ 1.5 and “poor” when it was < 1.5

### Calibration curve for L-Theanine and L-Theanine concentration profile in blood

**Fig. 2:**
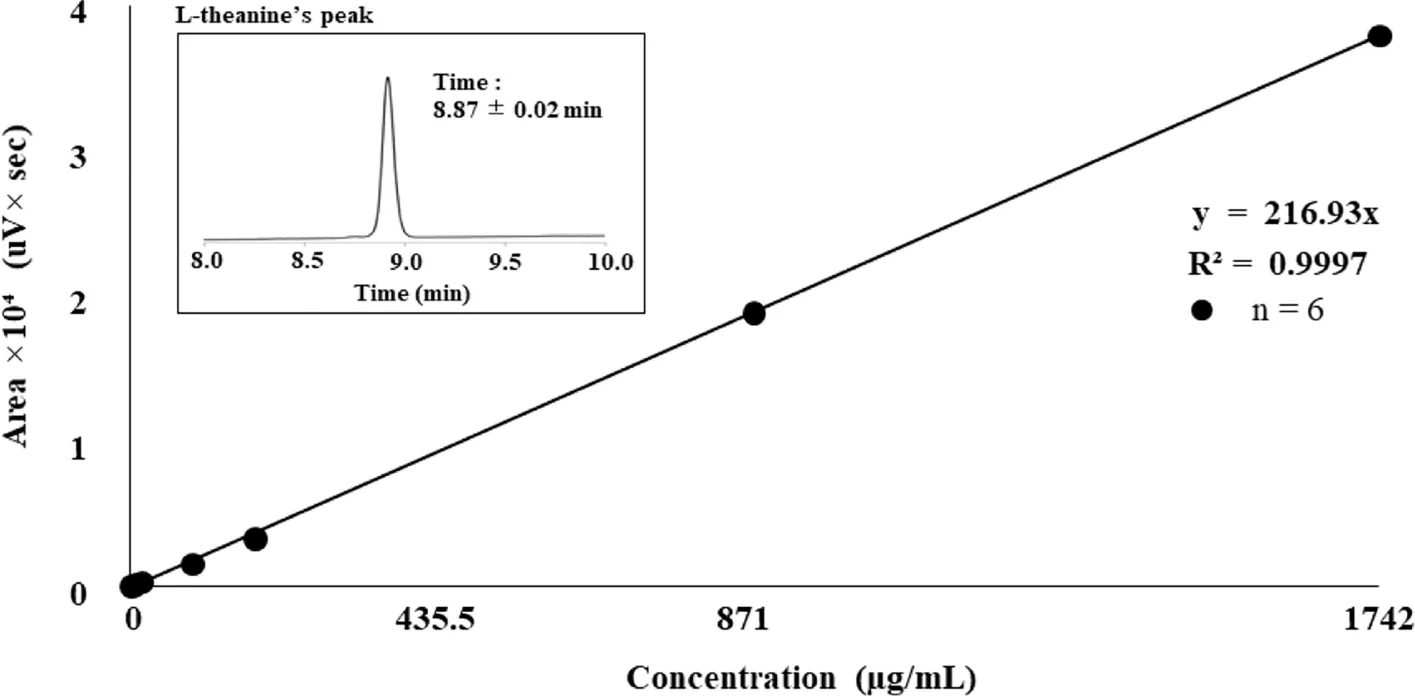
Calibration curve for measuring L-theanine using PITC precolumn derivatization and UV detection

The calibration curve for L-Theanine had excellent linearity in the concentration range of 1.74–1742.00 µg/mL, with an R^2^ value of 0.9997 and regression equation of y = 216.93x. (Fig. 2).

The coefficient of variation (%) was 1.56–4.75% at each concentration. The limit of detection for L-theanine was 0.44 µg/mL when the signal-to-noise ratio was set at 4 (Table 2).

**Table 2:** Standard deviation (SD) and coefficient of variation (CV; %) of L-theanine

Concentration (µg/mL)	Experimental Conc. (µg/mL)	SD	CV (%)
1.74	1.44	0.06	4.45
8.71	7.28	0.13	1.85
17.42	14.18	0.67	4.75
87.10	71.37	2.63	3.54
174.20	163.96	6.81	3.77
871.00	868.90	13.56	1.56
1742.00	1746.94	29.36	1.68

**Fig. 3:**
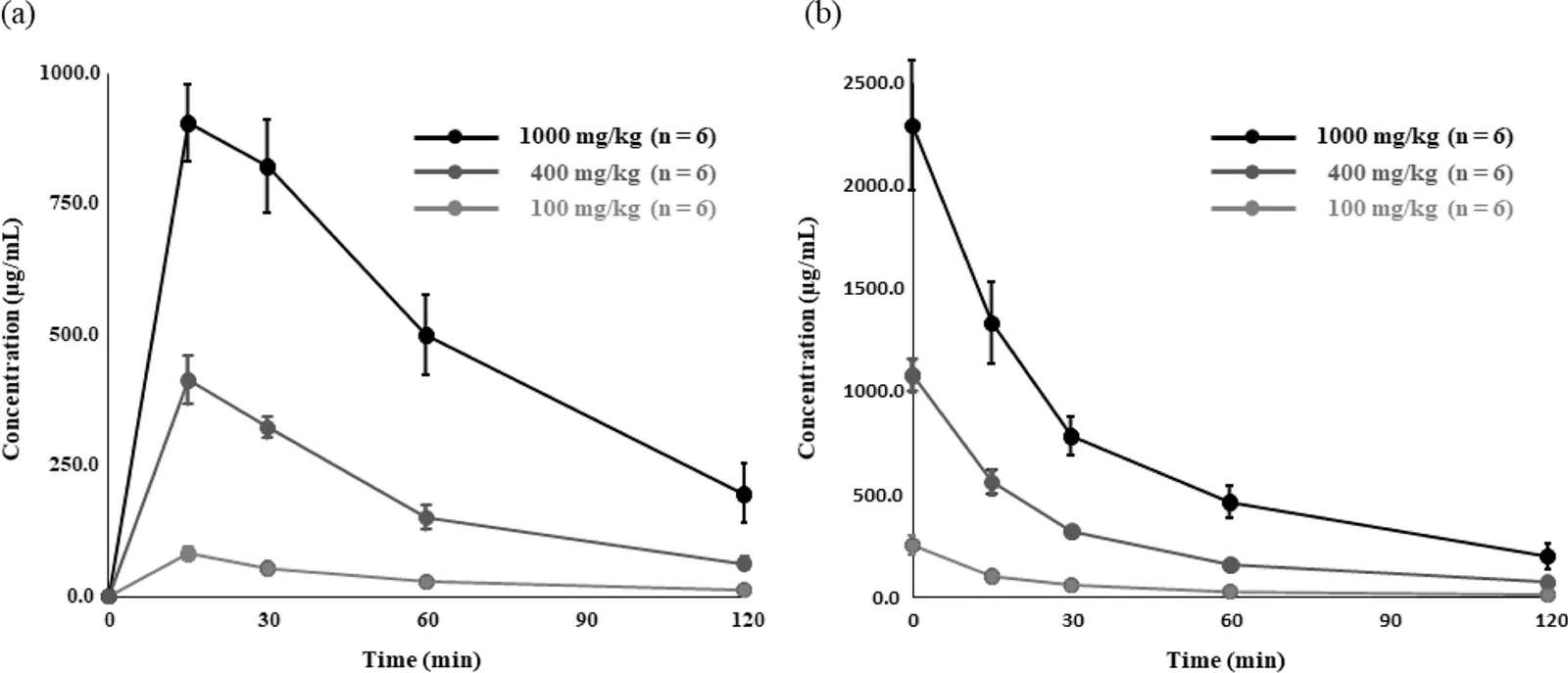
Serum concentrations of L-theanine administered orally (a) or via tail vein injection (b) at doses of 100, 400, and 1000 mg/kg

The concentrations of L-Theanine in oral administration were as follows: at the 100 and 400 mg/kg doses, the Tmax was 15 min post-dose and Cmax was 82.1 and 413.1 µg/ mL, respectively; at the 1000 mg/kg dose, the Tmax was 17.5 min post-dose and Cmax was 906.4 µg/mL. The concentrations at 120 min after administration were 11.46 ± 1.19, 61.94 ± 12.57, and 195.15 ± 57.04 µg/mL at doses of 100, 400, and 1000 mg/kg, and Cmax was 14.27 ± 3.04, 15.04 ± 2.96, and 21.40 ± 5.55%, respectively. When administered via tail vein injection, the concentrations of L-theanine at initial concentration( C_0_)were 253.04 ± 45.27, 1078.13 ± 79.47, and 2290.30 ± 315.55 µg/mL for doses of 100, 400, and 1000 mg/kg, respectively. The concentrations at 120 min after administration were 10.55 ± 1.03, 70.12 ± 15.44, and 197.37 ± 62.12 µg/mL at each dose, which decreased to the same level as the oral administration (Fig. 3).

### Pharmacokinetic parameters of L-Theanine

The pharmacokinetic parameters of L-theanine, including half-life (T_1/2_), CLtot, Vd, and disappearance rate constant (Ke), were determined for tail vein administration. T_1/2_ was 38.45 ± 3.87, 41.53 ± 4.46, and 46.50 ± 10.16 min at doses of 100, 400, and 1000 mg/kg, respectively. CLtot was 0.33 ± 0.06, 0.24 ± 0.02, and 0.24 ± 0.05 mL/min at doses of 100, 400, and 1000 mg/kg, respectively. The Vd values were 12.57 ± 2.15, 10.06 ± 0.77, and 11.03 ± 1.41 mL at doses of 100, 400, and 1000 mg/kg, respectively. Ke was 0.026 ± 0.002, 0.024 ± 0.002, and 0.022 ± 0.005/min at doses of 100, 400, and 1000 mg/kg, respectively. The AUC for L-theanine was 4.02 ± 0.39, 22.05 ± 1.88, and 60.34 ± 7.27 mg/mL･min at oral doses of 100, 400, and 1000 mg/kg, respectively, whereas the AUC for tail vein administration of L-theanine at 100, 400, and 1000 mg/kg doses were 6.17 ± 0.72, 32.77 ± 1.53, and 81.39 ± 8.20 mg/mL･min, respectively. From the AUC obtained after oral and tail vein administration, the bioavailability (BA) of L-theanine was approximately 65.22, 67.29, and 74.14% at doses of 100, 400, and 1000 mg/kg, respectively (Table 3).

**Table 3:** Kinetic parameters of L-theanine

		Administered orally mg/kg			Administered intravenously mg/kg		
		100	400	1000	100	400	1000
**Tmax**	**min**	15.0 ± 0.0	15.0 ± 0.0	17.5 ± 5.6	–	–	–
**Cmax(C0)**	**µg/mL**	82.10 ± 12.72	413.15 ± 44.62	906.44 ± 73.22	253.04 ± 45.27	1078.13 ± 79.47	2290.30 ± 315.55
**AUC (T)**	**mg/mL** **・min**	4.02 ± 0.39	22.05 ± 1.88	60.34 ± 7.27	6.17 ± 0.72	32.77 ± 1.53	81.39 ± 8.20
**T**_**1/2**_	**min**	–	–	–	38.45 ± 3.87	41.53 ± 4.46	46.50 ± 10.16
**CLtot**	**mL/min**	–	–	–	0.33 ± 0.06	0.24 ± 0.02	0.24 ± 0.05
**Vdss**	**mL**	–	–	–	12.57 ± 2.15	10.06 ± 0.77	11.03 ± 1.41
**Ke**	**/min**	–	–	–	0.026 ± 0.002	0.024 ± 0.003	0.022 ± 0.005
**F**	**%**	100		400		1000	
		65.22		67.29		74.14	

The parameters were measured at administrative doses of 100, 400, and 1000 mg/kg and n = 6. The parameters included time to maximum blood concentration (Tmax), maximum blood concentration (Cmax), area under the blood concentration–time curve (AUC), half-life (T1/2), total body clearance (CLtot), steady-state volume of distribution (Vdss), disappearance rate constant (Ke), and bioavailability (F).

### Chronological change in serum amino acid composition

**Fig. 4:**
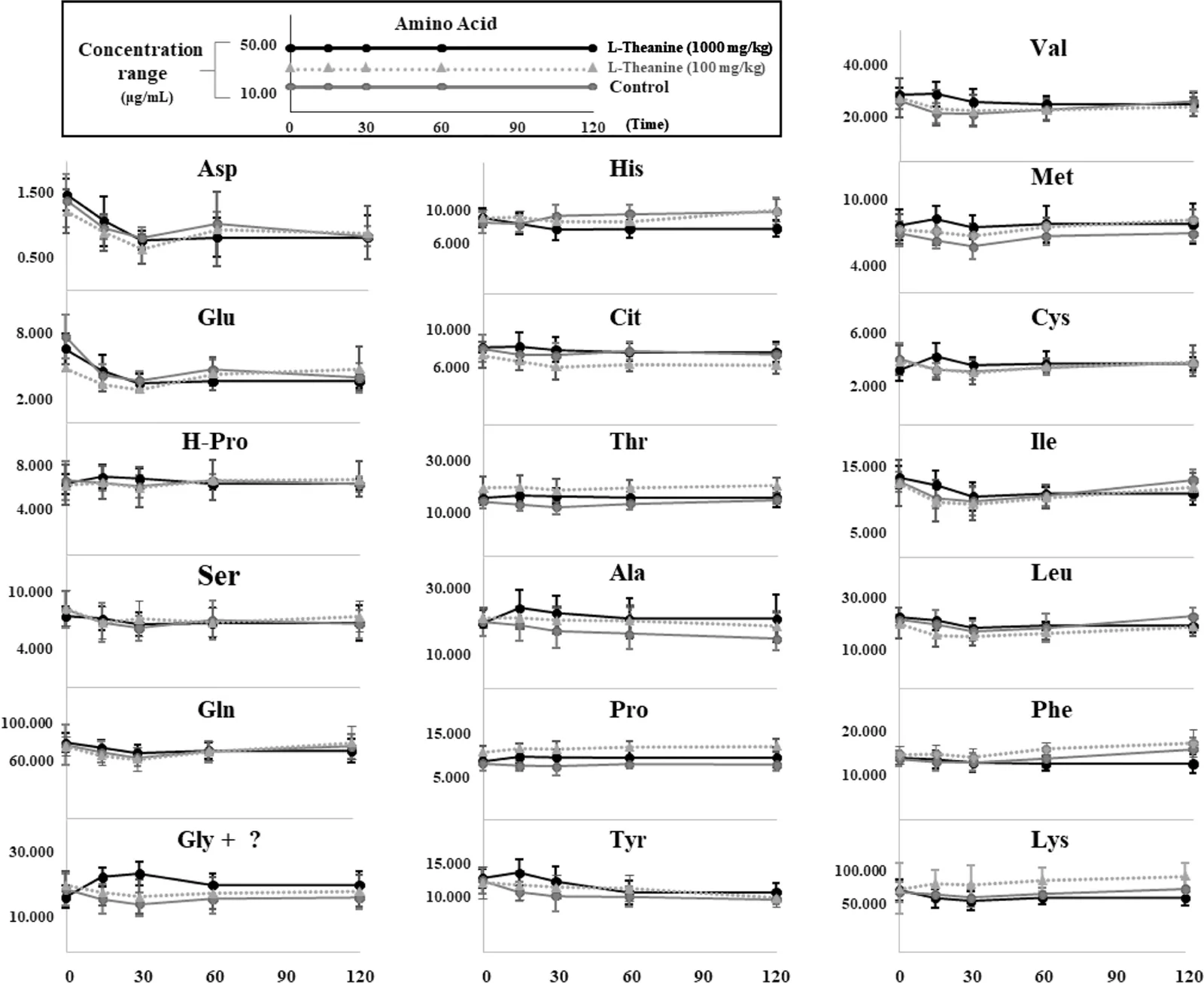
Changes in serum amino acid composition from 0–120 min in mice treated with saline (control) or L-Theanine (n = 6 per group)

**Fig. 5:**
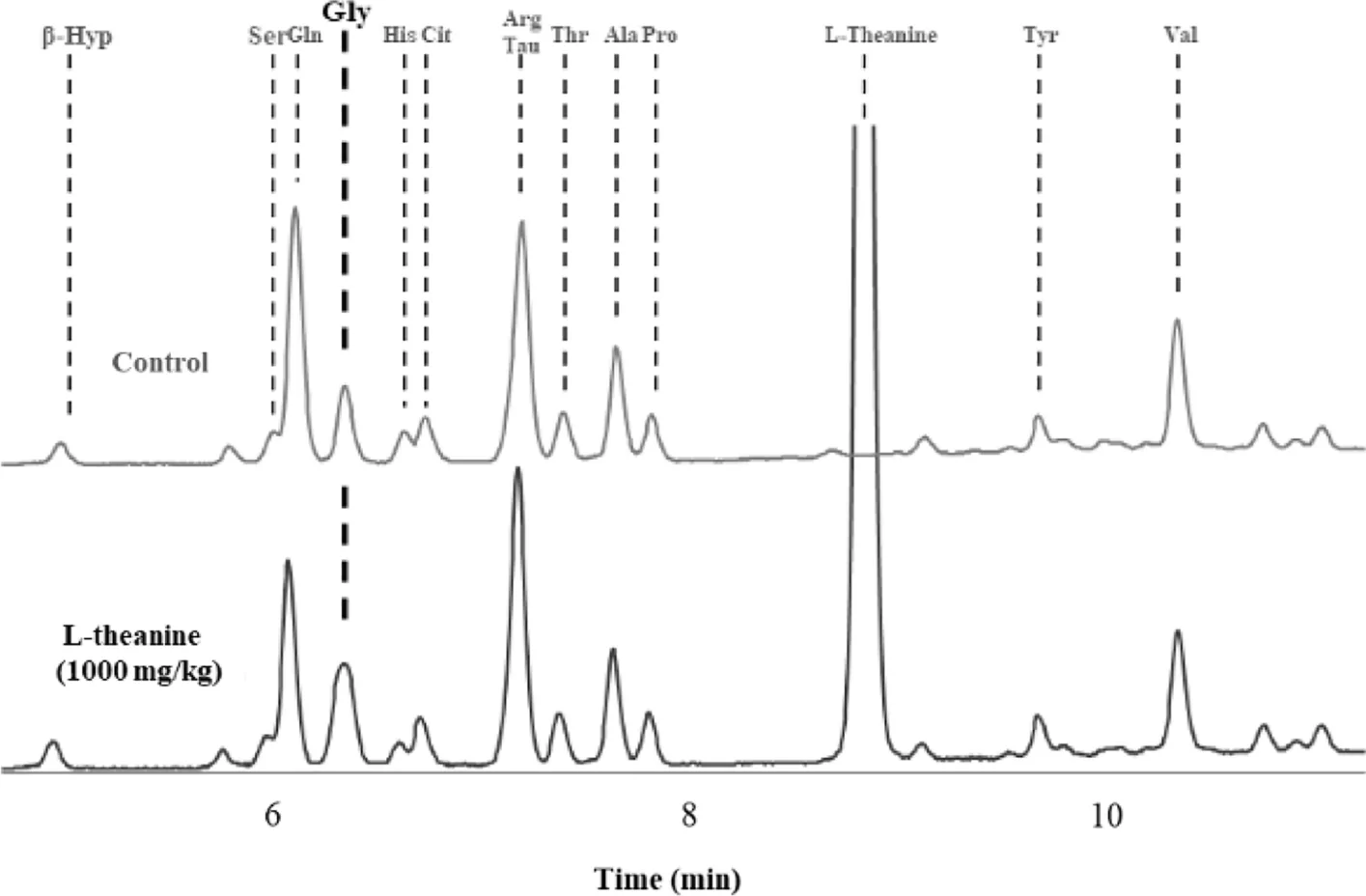
Peak of Glycine at 30 min after administration of 1000 mg of L-theanine compared to control

No significant changes compared to those of the control group were observed for the serum concentrations of Asp, Glu, β-Hyp, Ser, Gln, His, Cit, Ile, Leu, Phe, and Lys at 120 min for L-theanine doses of 100, 400, and 1000 mg/kg administered orally. Whereas, the concentrations of Thr, Ala, Pro, Tyr, Val, Met, and Cys were higher than those in the control group at 15–30 min after administration; however, the differences were not significant(Fig. 4). A significant increase in the peak area of Gly was observed at 15–30 min after administration of L-theanine at 400 and 1000 mg/kg; however, it may not be of a single Gly peak (Fig. 5).

## Discussion

This is the first study to apply the PITC method to detect L-theanine in biological samples and report the effect of L-theanine on pharmacokinetic parameters and serum amino acid composition in mice.

Among the several amino acid analyses, the separation and quantification of Glu using the PITC method has been reported to be superior to the O-phthaldehyde (OPA) labeling method (Fürst et al. 1990). Further, Thippeswamy et al. have reported that the L-theanine peak could be analyzed using the PITC method without interference from Asp, Glu, Ser, Gly, His, Arg, Thr, Ala, Pro, Tyr, Val, Met, Cys, Ile, Leu, Phe, or Lys (Thippeswamy et al. 2006). Additionally, there is another report that L-theanine in pu-erh and green tea could be analyzed using the PITC method and that other amino acids in tea leaf samples could be detected simultaneously (Zhu et al. et al. 2016); however, these two reports were not on biological samples such as blood. Therefore, there are some amino acids, such as Tau and Orn, that were not considered for isolation in these papers. Terashima et al. quantified L-theanine concentrations in rat blood; however, they used an automated amino acid analyzer with ninhydrin, and chromatograms were not shown and quantitation was not discussed (Terashima et al. 1999). Therefore, we adopted the PITC method to analyze L-theanine, including Glu, a degradation product of L-theanine. Furthermore, this study revealed that β-Hyp, Tau, GABA, Trp, and Orn, which have not been previously examined, did not interfere with the analysis of L-theanine. In this study, we isolated L-theanine without interference from the serum components and other 18 amino acids (Asp, Glu, Ser, Gly, His, Cit, Thr, GABA, Ala, Pro, Tyr, Val, Met, Cys, Ile, Leu, Phe, and Lys) that could be quantified simultaneously. However, Tau and Arg, and Orn and Trp, with the same retention times, could not be separated by this method. Therefore, the PITC method is an excellent analytical method that can separate and quantify L-theanine in biological samples to the limit of quantification of 0.44 µg/mL, suggesting its application for quantifying L-theanine in the brain and other tissues.

There have been reports of L-theanine being administered at doses ranging from 5 to 500 mg/kg in mice and 100 to 4000 mg/kg in rats (Sumathi et al. 2014; Takarada et al. 2015; Yokogoshi et al. 1998; Yuan et al. 2023). In the present tstudy, mice were administered L-theanine at doses of 100, 400, and 1000 mg/kg. Our findings indicate that the Cmax and AUC results of L-theanine oral administration to mice indicated that the blood concentration of L-theanine showed dose-dependent linearity. The average T_1/2_ of L-theanine, when administered orally to mice, was 42.16 ± 7.23 min. However, L-theanine is less likely to be eliminated than Arg, compared with the reported T_1/2_ of Arg in rats, which was 10.1 min (Campistron et al. 1982). Further, our results suggest that although L-theanine is rapidly absorbed, its elimination may be delayed at higher doses, such as 1000 mg/kg. A study on the pharmacokinetics of L-theanine in rats reported that at a dose of 4000 mg/kg administered intragastrically, the Tmax was reached 1 h post administration and the blood concentration of L-theanine decreased to approximately 15% or less of Cmax 16 h after administration (Terashima et al. 1999). However, they collected blood at 0, 1, 2, 5, 8, 16, and 24 h, and considering that a very high dose of L-theanine (4000 mg/kg) was administered, the Tmax may have been achieved earlier, resulting in the delayed disappearance of L-theanine. For sleep-improving effects, L-theanine supplements are often taken before sleep, and L-theanine reportedly takes approximately 30 min to elicit its effect (Kobayashi and Nagato 1998). Therefore, it is necessary to observe the changes in the blood concentration of L-theanine within 1 h after administration.

L-theanine has been suggested to be distributed not only in plasma but also in other tissues (Scheid et al. 2012), and the volume of distribution in mice in the present study suggested the same. Considering the body weight of the mice used in this study, it was suggested that although L-theanine is distributed in tissues, but its transferability is low.

Further, we have clarified the previously unknown bioavailability and clearance of L-theanine. Although the bioavailability of theanine in mice may differ from that in humans, it will be useful for future studies to elucidate the effects of theanine on brain function.

In the present study, mice survived well after tail vein administration at a dose of 1000 mg/kg, suggesting that the biological effects of L-theanine ingestion are mild and may not have a significant effect on serum amino acid composition. Terashima et al. reported that when L-theanine was administered to rats, the Trp concentration tended to decrease 24 h after administration, but other amino acid compositions in the blood did not change significantly within 24 h (Terashima et al. 1999). In this study, considering that investigating the immediate effect of L-theanine after administration is also important, we observed the effect of amino acid composition for 2 h after administration. Our results showed that the serum concentrations of Phe, Thr, Tyr, Met, Ala, Val, Leu, Ile, Lys, His, Glu, Gln, and Asp were unchanged 2 h after L-theanine administration, consistent with the results of Terashima’s report (Terashima et al. 1999). Notably, Trp was not compared as it could not be separated or quantified using the PITC method. We observed an increase in the Gly peak area 30 min after the oral administration of L-theanine at doses of 400 and 1000 mg/kg. This increase in peak area was significantly greater at the 1000 mg/kg dose than at the 400 mg/kg dose. However, this increase in the peak area was not sharp and could not be attributed to Gly alone. Based on the retention time of the peak, the possibility of ethylamine and ammonia, which are metabolites of theanine, was not considered. It has been reported that L-theanine may be converted to y-glutamylglycine in the presence of glycylglycine (Tsuge et al. 2003). Yamada et al. also reported that the administration of L-theanine to rats may induce inhibitory neurotransmission via glycine receptors by releasing Gly from brain neurons (Yamada et al. 2005). However, we were unable to determine whether the increase in this peak was owing to an increase in Gly alone. Given that Gly, similar to L-theanine, is used as a dietary supplement for its anxiolytic effects and to improve sleep, determining the effects of L-theanine administration on Gly levels is important and needs further research.

## Conclusions

In conclusion, we clarified the pharmacokinetics of L-theanine in mice weighing around 30 g as 10 ~ 12 mL for Vd and 0.24 ~ 0.33 mL/min for CLtot. Notably, the bioavailability of L-theanine was approximately 70%. Our study findings suggest that taking L-theanine as a supplement is safe without affecting its own levels or serum levels of other amino acids; however, further studies on Gly are needed.

## References

[CR1] Campistron G, Guiraud R, Cros J, Prat G (1982) Pharmacokinetics of arginine and aspartic acid administered simultaneously in the rat: II. Tissue distribution. Eur J Drug Metab Pharmacokinet 7(4):315–322. 10.1007/BF03189635, PMID: 7166183 10.1007/BF03189635 7166183

[CR2] Deng Y, Xiao W, Chen L, Liu Q, Liu Z, Gong Z (2016) In vivo antioxidative effects of l-theanine in the presence or absence of Escherichia coli-induced oxidative stress. J Funct Foods 24:527–536. 10.1016/j.jff.2016.04.029

[CR3] Fürst P, Pollack L, Graser TA, Godel H, Stehle P (1990) Appraisal of four pre-column derivatization methods for the high-performance liquid chromatographic determination of free amino acids in biological materials. J Chromatogr 499(499):557–569. 10.1016/s0021-9673(00)97000-6. (**PMID: 2324214**) 2324214 10.1016/s0021-9673(00)97000-6

[CR8] Jang HS, Jung JY, Jang lS, Jang KH, Kim SH, Ha JH, Suk K, Lee MG, (2012) L-theanine partially counteracts caffeine-induced sleep disturbances in rats. Pharmacol Biochem Behav 101(2):217–221 (**PMID: 22285321**) 22285321 10.1016/j.pbb.2012.01.011

[CR9] Kobayashi K, Nagato Y, AoI N, Juneja LR, Kim M, Yamamoto T, Sugimoto S (1998) Effects of L-Theanine on the Release of a-Brain Waves in Human Volunteers. Nippon Nogeikagaku Kaishi 72:153–157

[CR10] Li MY, Liu HY, Wu DT, Kenaan A, Geng F, Li HB, Gunaratne A, Li H, Gan RY (2022) L-theanine: A unique functional amino acid in tea (Camellia sinensis L.) with multiple health benefits and food applications. Front Nutr 9(9):853846. 10.3389/fnut.2022.853846, PMID: 35445053 10.3389/fnut.2022.853846 PMC9014247 35445053

[CR12] Scheid L, Ellinger S, Alteheld B, Herholz H, Ellinger J, Henn T, Helfrich HP, Stehle P (2012) Kinetics of L-theanine uptake and metabolism in healthy participants are comparable after ingestion of L-theanine via capsules and green tea. J Nutr 142(12):2091–2096. 10.3945/jn.112.166371. (**PMID: 23096008**) 23096008 10.3945/jn.112.166371

[CR13] Sumati T, Asha D, Suganya SN, Prasanna K, Sandhya KM (2014) Neuroprotective activity of L-theanine on 3-nitropropionic acid-induced neurotoxicity in rat striatum. Int J Neurosci 124(9):673–684 24325390 10.3109/00207454.2013.872642

[CR14] Takarada T, Nakamichi N, Kakuda T, Nakazato R, Kokubo H, Ikeno S, Nakamura S, Hinoi E, Yoneda Y (2015) Daily oral intake of theanine prevents the decline of 5-bromo-2’-deoxyuridine incorporation in hippocampal dentate gyrus with concomitant alleviation of behavioral abnormalities in adult mice with severe traumatic stress. J Pharmacol Sci 127(3):292–297 25837925 10.1016/j.jphs.2014.12.018

[CR15] Terashima T, Takido J, Yokogoshi H (1999) Time-dependent changes of amino acids in the serum, liver, brain and urine of rats administered with theanine. Biosci Biotechnol Biochem 63(4):615–618. 10.1271/bbb.63.615. (**PMID: 10361674**) 10361674 10.1271/bbb.63.615

[CR16] Thippeswamy R, Gouda MKG, Rao DH, Martin A, Gowda LR (2006) Determination of theanine in commercial tea by liquid chromatography with fluorescence and diode array ultraviolet detection. J Agric Food Chem 54(19):7014–7019. 10.1021/jf061715+. (**PMID: 16968057**) 16968057 10.1021/jf061715+

[CR17] Tsuge H, Sano S, Hayakawa T, Kakuda T, Unno T (2003) Theanine, γ-glutamylethylamide, is metabolized by renal phosphate-independent glutaminase. Biochim Biophys Acta 1620(1–3):47–53. 10.1016/s0304-4165(02)00504-4. (**PMID: 12595072**) 12595072 10.1016/s0304-4165(02)00504-4

[CR18] Unno K, Sumiyoshi A, Konishi T, Hayashi M, Taguchi K, Muguruma Y, Inoue K, Iguchi K, Nonaka H, Kawashima R, Hasegawa-Ishii S, Shimada A, Nakamura Y (2020) Theanine, the main amino acid in tea, prevents stress-induced brain atrophy by modifying early stress responses. Nutrients 12(1):174. 10.3390/nu12010174. (**PMID: 31936294**) 31936294 10.3390/nu12010174 PMC7019546

[CR19] Yamada T, Terashima T, Okubo T, Juneja LR, Yokogoshi H (2005) Effects of theanine, r-glutamylethylamide, on neurotransmitter release and its relationship with glutamic acid neurotransmission. Nutr Neurosci 8(4):219–226. 10.1080/10284150500170799. (**PMID: 16493792**) 16493792 10.1080/10284150500170799

[CR20] Yokogoshi H, Kobayashi M, Mochizuki M, Terashima T (1998) Effect of theanine, r-glutamylethylamide, on brain monoamines and striatal dopamine release in conscious rats. Neurochem Res 23(5):667–673. 10.1023/a:1022490806093 9566605

[CR21] Yuan H, Ling L, Kehong L, Enshuo L, Shumin H, Zhihua G, Wenjun X (2023) L-Theanine alleviates heat stress-induced impairment of immune function by regulating the p38 MAPK signalling pathway in mice. Food Funct 14(1):335–343 36511090 10.1039/d2fo02775e

[CR22] Zhang Y, Jia X, Chen X, Liu Y, Zhao Z, Hao J, Wu R, Feng H, Ren X (2021) L-theanine and Neumentix mixture improves sleep quality and modulates brain neurotransmitter levels in mice. Ann Palliat Med 10(4):4572–4581. 10.21037/apm-21-663, PMID: 33966405 10.21037/apm-21-663 33966405

[CR23] Zhao J, Zhao X, Tian J, Xue R, Luo B, Lv J, Gao J, Wang M (2020) Theanine attenuates hippocampus damage of rat cerebral ischemia-reperfusion injury by inhibiting HO-1 expression and activating ERK1/2 pathway. Life Sci 241(241):117160. 10.1016/j.lfs.2019.117160. (**PMID: 31837331**) 31837331 10.1016/j.lfs.2019.117160

[CR24] Zhu Y, Luo Y, Wang P, Zhao M, Li L, Hu X, Chen F (2016) Simultaneous determination of free amino acids in pu-erh tea and their changes during fermentation. Food Chem 194(194):643–649. 10.1016/j.foodchem.2015.08.054. (**PMID: 26471603**) 26471603 10.1016/j.foodchem.2015.08.054

